# Supplementary material for: Mechanisms governing the pioneering and redistribution capabilities of the non-classical pioneer PU.1
Source: Nat Commun. 2020 Jan 21;11:402. doi: 10.1038/s41467-019-13960-2 (PMC6972792; doi:10.1038/s41467-019-13960-2)
Supplement: Supplementary file 7 — Source data [file 41467_2019_13960_MOESM7_ESM.zip › Source_Data/Figure5/Figure5A_MotifScanOutput/homerResults/motif2.similar.html]

motif2

## Information for motif2

T
G
A
C
G
A
T
C
C
T
G
A
A
G
T
C
G
C
A
T
A
G
C
T
G
T
A
C
A
G
T
C
C
G
A
T
A
C
T
G
A
G
C
T
T
A
G
C
  
Reverse Opposite:  

A
T
C
G
T
C
G
A
A
G
T
C
C
G
T
A
A
C
T
G
A
C
T
G
C
T
G
A
C
G
T
A
C
T
A
G
G
A
C
T
C
T
A
G
A
C
T
G
  

|  |  |
| --- | --- |
| p-value: | 1e-181 |
| log p-value: | -4.170e+02 |
| Information Content per bp: | 1.652 |
| Number of Target Sequences with motif | 801.0 |
| Percentage of Target Sequences with motif | 26.56% |
| Number of Background Sequences with motif | 3696.3 |
| Percentage of Background Sequences with motif | 8.10% |
| Average Position of motif in Targets | 217.7 +/- 175.2bp |
| Average Position of motif in Background | 205.3 +/- 134.4bp |
| Strand Bias (log2 ratio + to - strand density) | 0.0 |
| Multiplicity (# of sites on avg that occur together) | 1.12 |
| Motif File: | file (matrix) reverse opposite |

### Similar de novo motifs found

|  |  |  |  |  |  |  |  |
| --- | --- | --- | --- | --- | --- | --- | --- |
| Rank | Match Score | Redundant Motif | P-value | log P-value | % of Targets | % of Background | Motif file |
| 1 | 0.965 | C T G A A G T C C G A T A C G T A G T C A G T C A C G T A C T G A G T C | 1e-168 | -387.179999 | 33.16% | 12.77% | motif file (matrix) |
| 2 | 0.954 | T C G A A G C T G C A T A C G T T G A C T G A C G A C T A C T G A G C T A T G C | 1e-165 | -380.622280 | 26.62% | 8.72% | motif file (matrix) |
| 3 | 0.939 | C A G T A T C G T G C A G T A C T C G A C A T G T A C G T G C A G C T A C T A G A G C T | 1e-148 | -342.693756 | 16.68% | 3.84% | motif file (matrix) |
| 4 | 0.866 | A G T C C G A T A C G T A G T C A G T C A C G T A C T G A G T C | 1e-123 | -284.992943 | 43.63% | 23.28% | motif file (matrix) |
| 5 | 0.917 | T G A C G A T C G A T C C G T A A G T C G C A T A G C T A G T C A G T C C G A T A T C G G A C T T A G C | 1e-119 | -276.087039 | 12.37% | 2.57% | motif file (matrix) |
| 6 | 0.942 | A G T C G T C A A C T G A C T G C G T A C G T A C T G A | 1e-90 | -209.150473 | 44.20% | 26.41% | motif file (matrix) |
| 7 | 0.938 | G C A T A G T C C T A G A G C T A C G T A C G T A T G C A G T C A C G T A C T G A G C T A G T C G A C T A G C T | 1e-81 | -187.683111 | 12.60% | 3.79% | motif file (matrix) |
| 8 | 0.751 | T G A C G T C A C T A G A C T G C T G A C G A T A C T G A G C T C T A G A C T G T A C G | 1e-65 | -150.072985 | 4.71% | 0.61% | motif file (matrix) |
| 9 | 0.790 | G C T A C A G T T G A C C G A T C T A G A G C T A G C T A C G T A G T C A G T C A C G T C A T G A G C T | 1e-32 | -74.772581 | 6.80% | 2.50% | motif file (matrix) |
